# Compared DNA and RNA quality of breast cancer biobanking samples after long-term storage protocols in − 80 °C and liquid nitrogen

**DOI:** 10.1038/s41598-020-71441-9

**Published:** 2020-09-01

**Authors:** Maximilian Babel, Andreas Mamilos, Stephan Seitz, Tanja Niedermair, Florian Weber, Tobias Anzeneder, Olaf Ortmann, Wolfgang Dietmaier, Christoph Brochhausen

**Affiliations:** 1 Institute of Pathology and Central Biobank Regensburg, University Regensburg, Franz-Josef-Strauß-Allee 11, 93053 Regensburg, Germany; 2 Department of Gynaecology and Obstetrics, University Medical Centre Regensburg, Regensburg, Germany; 3 Central Biobank Regensburg, University Clinic and University Regensburg, Regensburg, Germany; 4 Patients’ Tumor Bank of Hope (PATH-Biobank) Foundation, Munchen, Germany

**Keywords:** Diagnostic markers, Pathology, Cancer

## Abstract

Molecular investigations are crucial for further developments in precision medicine. RNA sequencing, alone or in combination with further omic-analyses, resulted in new therapeutic strategies. In this context, biobanks represent infrastructures to store tissue samples and body fluids in combination with clinical data to promote research for new predictive and prognostic biomarkers as well as therapeutic candidate molecules. Until today, the optimal storage conditions are a matter of debate especially with view to the storage temperature. In this unique approach we compared parallel samples from the same tumour, one half stored at − 80 °C and one half in the vapor phase of liquid nitrogen, with almost identical pre-analytical conditions. We demonstrated that RNA isolated from breast cancer samples revealed significantly higher RIN^e^-values after 10 years of storage in the vapor phase of liquid nitrogen compared to storage at − 80 °C. In contrast, no significant difference was found regarding the DIN-values after DNA isolation. Morphological changes of the nucleus and cytoplasm, especially in the samples stored at − 80 °C, gave insights to degenerative effects, most possibly due to the storage protocol and its respective peculiarities. In addition, our results indicate that exact point-to point documentation beginning at the sample preparation is mandatory.

## Introduction

In precision medicine and modern pathological diagnostics, molecular methods like gene expression analyses become more and more important to determine not only the origin of human diseases but also to clear-up prognostic as well as predictive factors and finally to find appropriate new therapeutic strategies^1,2^. In this context, the integrity of different omic-levels, namely the genome, epigenome, transcriptome, proteome and metabolome are of special interest to understand specific cellular mechanisms, which could be a target for clinical strategies^3^. Thus, new approaches of next generation sequencing (NGS) to detect mutations are in development^1,4^. On the level of the transcriptome, fast and precise fusion gene diagnosis can be made with help of RNA-sequencing, resulting in new, therapeutic treatment options such as the use of Crizotinib or Imatinib-mesylate^4–6^. With view to breast cancer, using microarray technology, four subtypes associated with patient response to chemotherapy have been defined based on a set of RNA patterns^7^. Furthermore, since a couple of years it is well known that miRNAs are important in disease development and progression through gene regulatory functionality^8,9^. Of special interest for tumour progression are the findings from Daugaard et al*.*, who identified miRNAs that were significantly associated with remote metastatic disease in lung adenocarcinoma^10^. Thus, RNA-sequencing became more and more important for molecular diagnostics and disease management often within a multi-omics or integrative manner. In this context, novel molecular subgroups of tumours associated with treatment response and survival could be characterized, namely for cholangiocarcinoma, oesophageal, pancreatic and prostate carcinoma^11–15^. The use of high-quality RNA or DNA is mandatory for all those molecular techiques^1,2,16^. Therefore, Kap et al*.* described four groups to define RNA quality, ranging from low quality with an RNA-integrity-number (RIN) below five, that where not reliable for any downstream analysis up to RIN values above eight, where all downstream techniques should give reliable results^16^. Besides, there are other classifications of RIN-values that suggest lower- or other cut-offs for good quality downstream analysis^17,18^. Nevertheless, we decided to use the classification of Kap et. al. due to its clear division into four RIN-depending quality groups^16^. In this context it is also documented that in general, extraction of nucleic-acids out of formalin fixed, paraffin-embedded samples result in lower quality of RNA due to higher fragmentation and modification^19^.

Hospital based biobanks are modern infrastructures for long-term storage of tissues and body fluids to support the concept of precision medicine and optimizing our understanding of the molecular mechanisms of diseases, mainly of malignant tumours and their progression. A major goal of biobanking is to find new candidates for innovative treatment options. For this purpose, high sample quality is crucial. In biobanks, samples can be stored natively, without formalin or paraffin for long-term storage. Considering this fact, it would be desirable to reach RIN-values above eight from samples that were stored in biobanks to generate the most reliable RNA-based data. To reach this goal, the extensive knowledge which has been developed by research driven biobanks should be adopted by clinical biobanks^20^. In this context, several studies revealed no influence of storage temperature or storage time on sample quality^21–23^: for long time storage in the vapour phase of liquid nitrogen (in the following and according to Auer et al*.* referred to as VPLN^24^), Kelly et al*.* analysed RIN and DIN values from 87 samples out of 14 different cases. Storage time did not affect RNA and DNA quality from samples that were stored for at least 10 years. Therefore, they stated, that in the VPLN samples can be preserved in good quality for up to 11 years^21^. Andreasson et al. found no time-dependent decrease of RIN-values for 153 tissue samples of different endocrine tissues (e.g. pheochromocytomas or thyroid cancers) that where stored at − 80 °C for up to 28 years. They also performed a morphological assessment with two pathologists who revealed no negative affect on sample quality^22^. Equal findings were given for gastric cancer tissue stored in − 80 °C for up to 12 years^23^. However, none of these studies compared neither RIN- or DIN-values nor the histological changings in tissues out of the same sample, stored at two different temperatures. This study aims to compare RIN equivalents (RIN^e^, algorithm based calculation, Agilent TapeStation 4200)- and DIN-values from the same tumour tissue stored for 10 years under two different storage temperatures, namely under − 80 °C and in the VPLN corresponding to about − 186°C^25^.

## Results

### RNA and DNA quality after storage at − 80 °C and in the VPLN

Correlating our RIN^e^ values to the four RNA “fit-for-purpose” quality groups stated by Kap et al*.* our results can be split up as follows^16^: from the total of 16 specimens stored at − 80 °C, one sample was below the cut-off of < 5 and therefore not reliable for downstream analysis. RIN^e^-values of two samples ranged between ≥ 5 and < 6 (appropriate for RT-qPCR). RIN^e^-values of eight samples ranged between ≥ 6 and < 8 (suitable for gene array analysis). RIN^e^-values ≥ 8 (suitable for all downstream techniques) were found in five samples.

Grouping the RIN^e^-values originating from the 16 parallel samples of the same tumour stored in the VPLN showed the following results: there was no sample with a RIN^e^-value below 5 and one sample with a RIN^e^-value between ≥ 5 and < 6. We found two samples between RIN^e^-values ≥ 6 and < 8. Most samples (13) correlated to the highest “fit-for-purpose” quality group of RIN^e^-values ≥ 8. The results are summarized in Table 1.

**Table 1:** Correlation of the RIN^e^-values into the RIN “fit-for-purpose” quality groups described in Kap et al.^16^.

Storage condition	“Fit-for-purpose” quality group			
	Number of samples with RIN^e^ < 5 (percentage)	Number of samples with RIN^e^ ≥ 5 and < 6 (percentage)	Number of samples with RIN^e^ ≥ 6 and < 8 (percentage)	Number of samples with RIN^e^ ≥ 8 (percentage)
80 °C	1 (6.25%)	2 (12.5%)	8 (50%)	5 (31.25%)
VPLN	0 (0%)	1 (6.25%)	2 (12.5%)	13 (81.25%)

Of all samples stored at − 80 °C, 81.5% reached RIN^e^-values of ≥ 6.0 but only 31.25% of the samples were found correlating to the highest fit for purpose group with RIN^e^-values ≥ 8.0. The mean RIN^e^-value from samples stored at − 80 °C was 7.14. In contrast, 93.75% of samples that were stored in the VPLN reached RIN^e^-values of ≥ 6.0. Also, 81.25% of all samples were found in the highest fit for purpose group with RIN^e^-values of ≥ 8.0 The mean RIN^e^-value of samples stored in the VPLN was 8.59. Statistical analysis of the means of the RIN^e^-values revealed significantly lower RIN^e^-values in samples stored at − 80 °C compared to samples stored in the VPLN (p = 0.0024, student’ s t test).

For the DNA, the mean DIN-value from the samples stored at − 80 °C was 6.99. The mean of samples stored in the VPLN was 7.42. According to the students t test, no significant differences could be observed in our samples (p = 0.21).

Pearsons product moment correlation coefficient (r) for comparison between RIN^e^ and DIN for the samples stored at − 80 °C was − 0.33. For the samples stored at VPLN, r was − 0.19.

**Figure 1:**
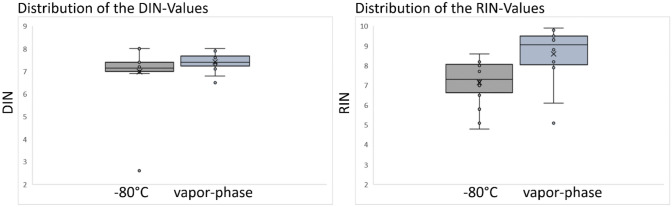
Boxplots of the distribution of the DIN- and RIN^e^-values, including statistical outliers and the median.

The RIN^e^- and DIN-values, including the 28 s/18 s Ratio and the RNA concentration of each sample is shown in Table 2. The results shown in Table 2 are demonstrated as a Boxplot in Fig. 1.

**Table 2:** RIN^e^- and DIN-values, concentration and 28 s/18 s ratio found in the 32 samples of 16 patients, stored under different conditions (− 80 °C/ VPLN).

Patient	Storage condition	28 s/18 s	RNA-concentration (ng/l)	RIN^e^-value	DIN-value
1	− 80 °C	0.9	62.4	**7.3**	**7.4**
	VPLN	1.3	39.8	**8.2**	**7.4**
2	− 80 °C	0.7	6.25	**5.1**	**8**
	VPLN	1.3	15.2	**5.1**	**7.9**
3	− 80 °C	0.9	107	**7.7**	**2.6**
	VPLN	2.7	133	**9.6**	**7.6**
4	− 80 °C	1.2	116	**7.3**	**6.9**
	VPLN	2.3	170	**9.8**	**7.6**
5	− 80 °C	0.7	48.2	**7.3**	**7.3**
	VPLN	2	438	**9.4**	**6.5**
6	− 80 °C	0.7	75.6	**6.5**	**7.1**
	VPLN	1.5	54.8	**8**	**7.3**
7	− 80 °C	1.1	31	**7.1**	**7**
	VPLN	1.8	47.1	**8.3**	**7.4**
8	− 80 °C	1.3	64.8	**8.2**	**7.1**
	VPLN	1.4	26.3	**8.8**	**7.1**
9	− 80 °C	0.8	62.9	**7**	**7.2**
	VPLN	1.7	78.7	**9.3**	**7.9**
10	− 80 °C	0.8	179	**7.2**	**7.1**
	VPLN	2.3	248	**8.8**	**7.6**
11	− 80 °C	1	27.4	**4.8**	**8**
	VPLN	1.7	70.5	**9.3**	**7.3**
12	− 80 °C	1.3	47.1	**8.1**	**7**
	VPLN	2.2	97	**9.5**	**7.7**
13	− 80 °C	0.8	38	**5.8**	**7.5**
	VPLN	2.8	29.7	**9.5**	**8.0**
14	− 80 °C	1.1	176	**8.3**	**7**
	VPLN	–	2.69	**6.1**	**7.4**
15	− 80 °C	1.7	133	**8.6**	**7.2**
	VPLN	2.2	29.9	**7.9**	**7.2**
16	− 80 °C	0.9	64	**8**	**7.4**
	VPLN	3.3	177	**9.9**	**6.8**

### Histological changes after storage at − 80 °C and in the VPLN

**Figure 2:**
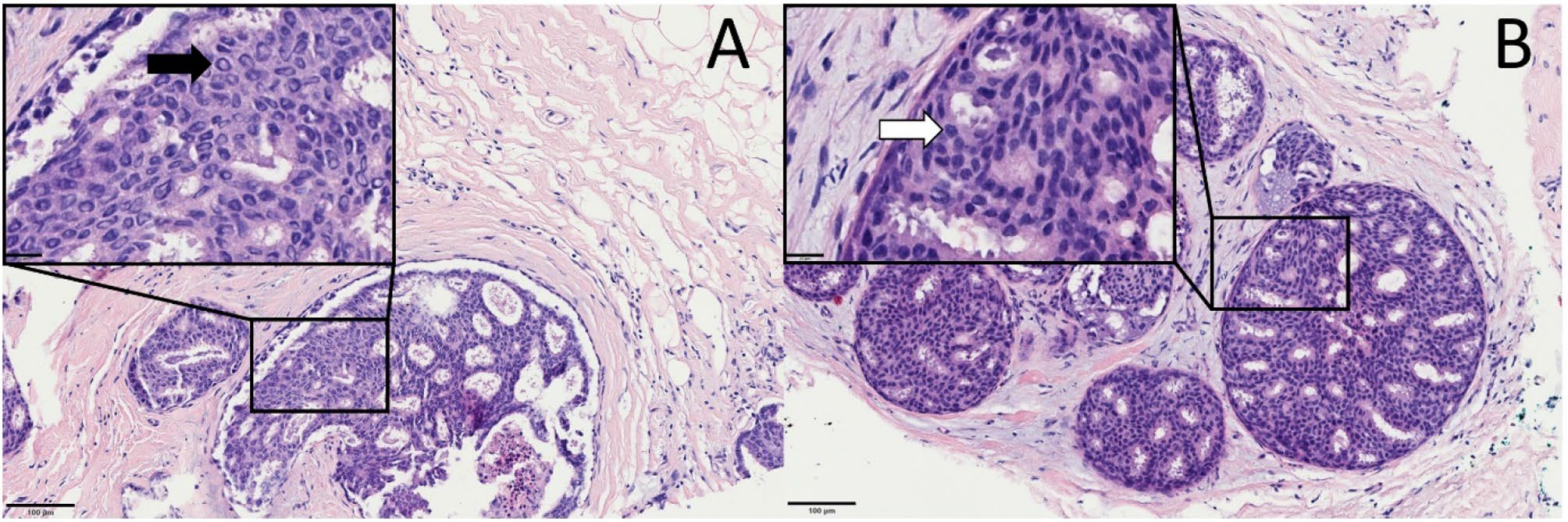
H&E-staining from Patient 3. (**A**) Sample stored at − 80 °C. (**B**) Sample stored in the VPLN. The major difference was given by brightening of the nuclear centre (Box **A**, black arrow) in contrast to the normal staining behaviour in the sample stored in the VPLN (Box **B**, white arrow). Also, distinct retraction artefacts at the interface to the fibrous tissue could be found more often in samples stored at − 80 °C. (H&E-staining, magnification ×200, scale-bar is 100 µm. Boxes: digital magnification, ×110, scale-bar is 20 µm).

To check a potential morphological correlation to these findings we performed H&E-staining of all samples and compared them. We found significantly abnormal nuclei in 11 out of 16 specimens stored at − 80 °C and in 2 out of 16 samples stored in the VPLN: the outer area of the nucleus was stained in dark blue with an inner, much brighter area which reacts more eosinophilic. Furthermore, hyperchromasia of the nuclei within the tumour cells was much more intensive in samples stored in the VPLN. Figure 2 shows a representative H&E-staining, which revealed the main difference given in an abnormal staining of the nuclei. Other differences were subtle in the conventional histological analyses: by help of H&E-staining cell–cell-borders seemed to be less clear in the -80 °C stored samples compared to the VPLN stored samples. Retraction-artefacts of the tissue were found in both H&E-stained sections, however with a higher extent in the -80 °C stored samples, especially at the interfaces between ductal epithelial cells and fibrous tissue (Fig. 2).

### Electron-microscopical analysis

**Figure 3:**
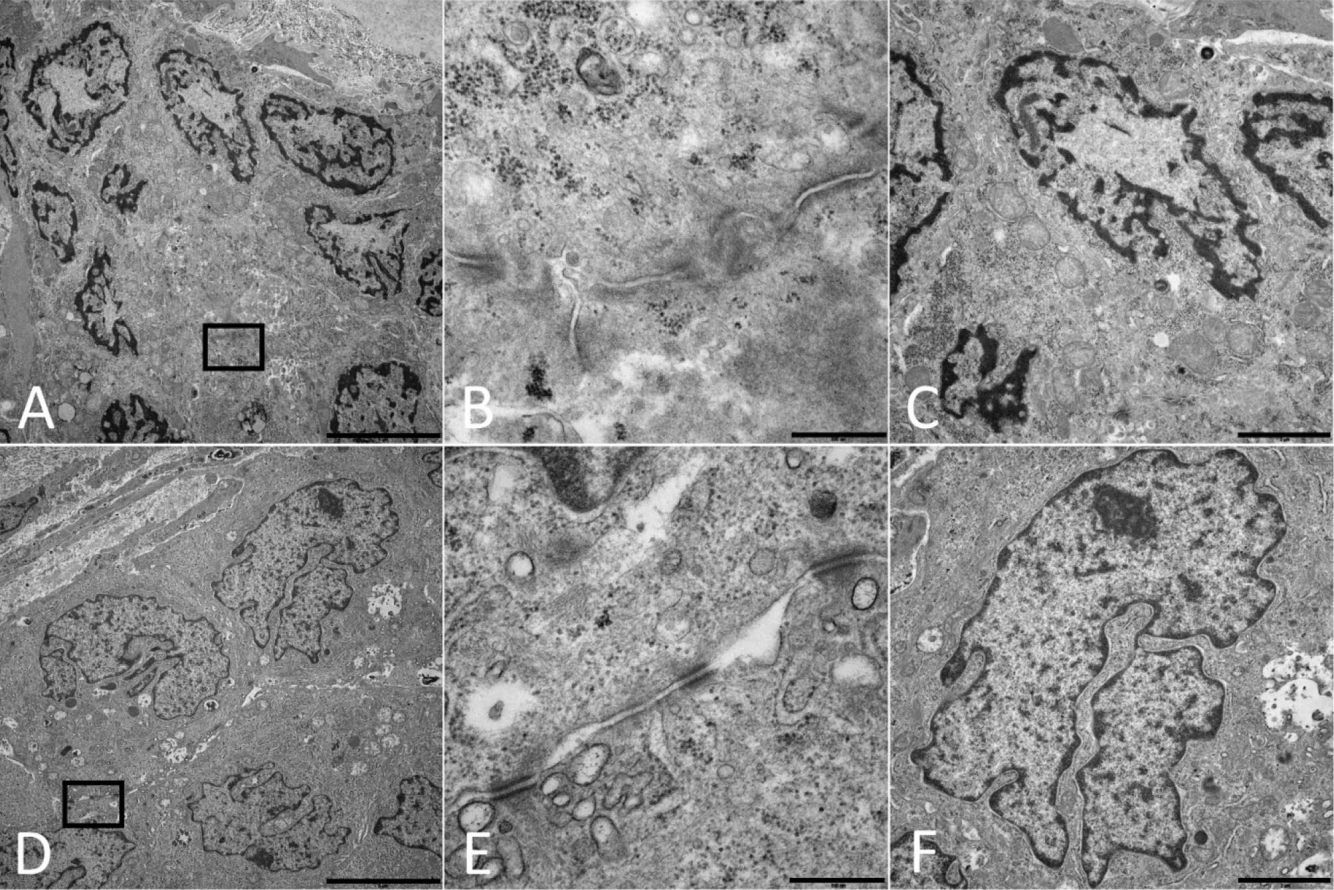
(**A**–**C**) Transmission electron microscopy from patient 10 stored at − 80 °C. (**A**) Group of tumour-cells (magnification ×5,000; scale-bar 5 µm). Black Box corresponds to (**B**) area shown in higher magnification. No clear-cell–cell borders can be found, also desmosomes appeared diffuse with poor contrast to the cytoplasm (magnification ×40,000, scale-bar 500 nm). (**C**) Higher magnification of a nucleus shown in (**A**). Less electron dense debris accumulated in the nuclei (magnification ×10,000, scale-bar 2 µm). (**D**–**F**) Transmission electron microscopy from patient 10 stored in the VPLN. (**D**) Group of tumour-cells (magnification ×5,000, scale-bar 5 µm). Black Box corresponds to (**E**) area showed in higher magnification. Well detectable cell–cell borders with intact membranes and visible desmosomes (magnification ×40,000; scale bar 500 nm). (**F**) Higher magnification of a nucleus shown in (**D**) (magnification ×10,000, scale-bar 2 µm).

To analyse potential ultrastructural correlates of the changes within the nuclei and the cell membranes, we performed electron-microscopy within exemplary samples. In transmission electron microscopical analyses tumour cells stored at − 80 °C revealed incomplete or no detectable cell–cell-borders, demonstrating a relevant missing of cell membranes. In contrast, specimens stored in the VPLN showed clearly detectable cell membranes. Furthermore, under storing conditions of − 80 °C, desmosomes appeared more diffuse with poor contrast to the cytoplasm than in specimens stored in the VPLN (Fig. 3, Part B). Also, we could find abnormal chromatin-distribution in the samples stored at − 80 °C. As a morphological correlate to the bright, eosinophilic centre of the nuclei in the H&E staining, we found high amounts of less electron dense deposits in the samples stored at − 80 °C.

## Discussion

According to our literature review, this paper represents the first report comparing the influence of the storage temperature on RIN^e^- and DIN-values from simultaneously obtained, natively stored tumour samples after long-term storage for at least 10 years at − 80 °C or in the VPLN. Additionally, results were enriched by histomorphological and electron-microscopical data.

Our findings demonstrate that RIN^e^-values are significantly lower in − 80 °C stored tumour samples compared to the ones stored in the VPLN. These findings are of special interest since both samples were obtained from the same tumour-resection as parallel specimens. Therefore, both biobank-specimens underwent the almost identical pre-analytical treatment. The fact of significantly lower RIN^e^-values in − 80 °C stored tissue specimens is of special relevance since new methods in precision medicine and molecular pathology are based on nucleic-acid-based techniques, such as RNA-sequencing^1,4,16^. Thus, high RNA-quality is mandatory to receive reliable analytical results and potential interpretations. With view to data interpretation for disease managing Lightbody et al*.* demonstrated the importance of the interplay of different omic-levels for future progress in research and clinical application^3^. With respect to high throughput analyses the sample quality is crucial not only for proper analyses of the transcriptome but also for other levels of omics, which are the genome, epigenome, proteome and metabolome. Regarding the transcriptome, Kap et al. described a RIN-value of 8.0 as a cut-off for excellent RNA^16^. Contrary to the Bioanalyzer used by Kap et al., the TapeStation 4200 we used applies an algorithm which calculates the RIN-equivalent RIN^e^^16^. But, according to Agilent, RIN and RIN^e^ show a high correlation, so one can assume that the results should be at least comparable^25^. In addition, RIN-values are based on fragmentation of rRNA, which is only one of many different RNA-species in a cell^26^. Therefore, results need to be considered more in detail when downstream analyses using other RNA-species are planed out of stored samples. Auer et al*.* found, that storage of human tissues at − 80 °C provided at least the same or even higher RIN-values than VPLN in 49 different tissue samples^24^. One potential explanation for the discrepancies between our studies and the ones in the literature could be that we used parallel specimen from the same tumour^21–23^. Using the same tumour entity but from different samples might not include individual peculiarities such as necrosis and different cellularity. Therefore, the fact that only one tumour entity was used in the present study could reinforce this discrepancy. Further studies are needed to clear-up this phenomenon in detail. Nevertheless, higher RIN or RIN^e^ values should result in better downstream analyses. In our analyses, the breast-cancer samples stored for 10 years in the VPLN reached higher rRNA-quality—estimated by RIN^e^. With view to the quality of DNA we could confirm the results of other scientists that found no difference in DNA-quality after long time storage^21^*.* A correlation between the behaviour of DNA and RNA-quality showed a medium negative correlation between DIN and RIN^e^ for the samples stored at − 80 °C. Only small negative correlation of DIN and RIN^e^ for the samples stored at the VPLN was found.

Beside the molecular differences in RNA-quality we identified morphological changes using standard histological techniques. In this context, our observation of central brightening in nuclei with eosinophilic inclusions was an interesting finding predominantly in − 80 °C stored samples. These inclusions correlate ultrastructurally with less electron dense debris in the nuclei. The fact that these findings were predominantly present in -80 °C stored samples indicates that this feature represents an artefact due to storing conditions, especially since the samples were obtained from the same tumour in the same session. This should be taken into consideration when stored tissue samples undergo histopathological analysis, because these artefacts could be misinterpreted as nuclear eosinophilic inclusions or pseudoinclusion respectively, which represent a pathological feature in morphological diagnostics^27^. In general, nuclear inclusions represent the presence of foreign material in the nucleus such as viral particles. In contrast, pseudoinclusions represents herniation of the cytoplasm in the nucleus, which is typically for papillary thyroid carcinoma and meningioma and was also described as a feature for neuroendocrine tumours of the lung, in the latter partly as beta-Catenin accumulation due to a mutation in the CTNNB1 gene^28,29^. Especially in cytopathological diagnostics nuclear inclusions and pseudoinclusions are of special relevance as diagnostic features^30^. Furthermore, in pathologic changes of the breast, intranuclear inclusions are described as a feature of benign lesions^31^. Taking these facts together, beside the relevant quality impairment for important molecular downstream analyses, storage conditions might result in morphological changes with impact on the histomorphological interpretation.

To analyse the underlying structural changes more in detail we performed transmission electron microscopy in representative samples, which revealed relevant changes not only in the cell nucleus given as a untypical chromatin distribution and potential oedema but also in the cytoplasm and the cell membranes. In this context, the higher degradation of desmosomes and cytoplasm in the − 80 °C stored tissue indicates relevant degenerative effects within this storage protocol. Interestingly, recent experimental studies on nuclear inclusions in the nervous system indicate their potential role in RNA degradation due to degenerative processes^32,33^. A further study has identified cytoplasmic inclusions in cells of the nucleus caudatus, immunohistologically positive for stress granule markers, indicating their potential role in cellular stress reaction^34^. In future studies we will analyse parallel samples with view to molecular and morphological stress markers to better understand the mechanisms behind the relevant RNA impairment under − 80 °C storage condition.

Combining the reduced RIN^e^-values with the morphological changes in the tissue and related literature, a higher rate of cell-damage is obvious in − 80 °C stored samples. One explanation for this phenomenon could be the metabolic activity of cells and tissues even under temperatures around and below − 80 °C. This has been shown in several experimental trials: Rasmussen et al*.* showed that Ribonuclease A does not lose its function until below − 53,15 °C, whereas Rouy et al. found 1% remaining activity of matrix metalloproteinase-9 stored at − 80 °C after 43 months because of degradation. Further examples are given in the review of Hubel et. al.^35–37^. However, there may be additional factors influencing the samples: given by the storage system such as ice crystal formation resulting from opening-cycles of a − 80 °C freezer device, which is not only visible at the device itself but which also damage intracellular structures^38^. In this context, it should be mentioned that the storage device for − 80 °C samples was a general tissue biobank freezer with various, several thousand samples that were used for many studies. Therefore, the freezer has been opened many times over the past 10 years to store samples or to remove others. Furthermore, there was no individual temperature monitoring for each sample and samples were not distributed evenly in the freezing system (e.g. some samples are stored near the door, whereas other samples where stored in the back of the freezer, near the compressor). Even if the strict measurement of the freezers core-temperature showed no deviation from the target-temperature of − 80 °C, these facts might have increased the temperature in at least a part of the samples. In contrast, the biobank that stored the samples in the VPLN was used just for breast-cancer-samples, resulting in a smaller number of different studies and with that a lower number of overall samples. As a result, there were fewer cycles of opening the storage device with fewer chances of increasing the temperature. Additionally, the samples for this study were stored in only two different, adjacent collecting-boxes on the same level within the freezing system.

Concerning all these facts it is more likely, that temperature fluctuations were more present at − 80 °C and might have negatively influenced the samples. One possible effect could be the formation of ice crystals within the tissue: the glass transition temperature (Tg) is the temperature, where the freezing process of a liquid needs to be fast enough to avoid crystal formation but results in an amorphous substance^39^. This effect is used in vitrification processes for cryo-conservation without damaging ice crystals^40^. For our studies Tg can only be estimated, but Meneghel et al. demonstrated a Tg around − 47 °C for human T-cells in suspension ^41^. Although our cells were not in suspension and no T-cells, cytoplasm might be of similar composition. So, one can estimate Tg around this temperature range. Re-freezing samples because of temperature fluctuations with slower cooling rates from above Tg back to storage temperature might result in ice crystals within the samples. A higher amount of ice crystals at the drawers of our − 80 °C freezer in contrast to the ones of the VPLN storage system supports this assumption. Corresponding ice crystal formation within the samples due to temperature fluctuations might be also responsible for the structural and molecular changes we found^40^. Regarding this fact, Germann, A. et al*.* could show that temperature fluctuations during various steps within the cryopreservation can negatively affect the biological quality of a sample^42^. All these technical aspects should be addressed in further studies as a potential influencer for tissue quality and emphasize the need for a well-developed documentation system also containing the freezing system itself.

With respect to the study design, one disadvantage might be the small number of sample-pairs. Therefore, future studies should compare a greater number of parallel samples. In contrast, a special strength of the present study in contrast to many other studies of this field, that the compared samples were obtained from the same tumour and surgical intervention and with that, same pre-analytical conditions^21–23^. In this case, our findings indicate that storing samples with an VPLN—protocol results in better RIN^e^ values and better morphological features. The latter will be analysed systematically in the future.

Considering all these facts and respecting the differing results of several other studies, it is likely that further, potentially yet unknown parameters in different freezing and storing protocols may influence the quality of stored tissue^21–23^. Nevertheless, we found differences in the RNA-quality with significantly higher RIN^e^-values in samples stored by the VPLN-protocol compared to the ones stored with an − 80 °C protocol after 10. These findings give evidence that tissue banking under VPLN conditions with as few as possible disturbances of storage conditions might be more suitable for molecular and structural downstream analyses in the cadre of precision medicine. Finally, it becomes clear that an exact point-to point documentation of preparation and storage is mandatory to enable proper analyses of the effects of preanalytical and storage issues for a better understanding of their effects on the biological quality of biobank specimens.

## Material and methods

### Tissue samples

The ethical review board of the University of Regensburg approved this study (Ref-number: 19-1408-101). The tissue samples were obtained from two local Biobanks located in the Institute of Pathology of the University Hospital of Regensburg: the PATH Biobank of Hope (hereinafter referred to as PATH-Biobank) and the general tumour bank of the Hospital St. Josef in Regensburg (hereafter referred to as tumour bank, also located in the Institute of Pathology of the University Hospital of Regensburg). Both biobanks are located in the same building at the Institute of Pathology of the University Regensburg in two neighbouring rooms. In the tumour bank, the samples were stored at − 80 °C (HFU-686, Thermo-Scientific, Waltham, USA). In the PATH-Biobank, the samples were stored in the VPLN at approx. − 186 °C (K-Series, Model 10, Worthington Industries). Every biobank stored several hundred samples of breast cancer. After comparison of the biobank-data we found 16 patients, where parallel samples from each patient were processed in the same way and were stored at the same day in the two different biobanks. Each pair of samples was stored for at least 10 years, with a range between 10.0 and 12.2 years. Transportation from the surgical theatre at the clinic in Regensburg to the Institute of Pathology of the University Regensburg was done at room temperature. At the institute, specimens for the routine diagnostics were taken immediately. In the same step, two representative parts of each tumour were taken and snap frozen in liquid nitrogen in separate tubes. Overall process took 5–10 min from sample arrival until snap freezing. Samples for the PATH-Biobank were transferred to a cryotube and stored immediately at VPNL in a freezer device, located at the Institute of Pathology in Regensburg. Samples for the Tumour-Bank where transferred into sterile plastic bags (3 × 5 cm) and stored at the same time in a − 80 °C freezer device in the same building. Transportation to the respective freezing system was done in liquid nitrogen. For at least 6 samples it was not possible to calculate the transportation-time (patient Nr. 1, 3, 4, 10, 13, 15), which were defined as not applicable with view to the transportation time (summary is given in Table 3).

**Table 3:** Description of the 16 sample-pairs.

Patient	Date of storage general Tumour bank	Date of storage PATH-Biobank	Transportation-time (min)	Weight (g) and measurements of excised tissue (cm)
1	23.08.2007	23.08.2007	Not applicable	44.5 g 4.0 × 4.0 × 3.0
2	27.08.2007	27.08.2007	35	61.7 g 6 × 6.2 × 4.5
3	18.09.2007	18.09.2007	Not applicable	26.6 g 4.5 × 4.2 × 2.5
4	11.10.2007	11.10.2007	Not applicable	80 g 8 × 7 × 6
5	15.10.2007	15.10.2007	64	530 g 18.5 × 13.5 × 6.5
6	23.10.2007	23.10.2007	8	23 g 6 × 5 × 3
7	05.11.2007	05.11.2007	56	49 g 8 × 6 × 3
8	21.11.2007	21.11.2007	8	56 g 5.5 × 5.5 × 2.5
9	30.01.2008	30.01.2008	60	140.7 g 9 × 7 × 6
10	07.03.2008	07.03.2008	Not applicable	576 g 22 × 14 × 7
11	12.03.2008	12.03.2008	127	344 g 20 × 15 × 3
12	16.05.2008	16.05.2008	55	283 g 17.5 × 12.9 × 8
13	10.06.2008	10.06.2008	Not applicable	69 g 11 × 6 × 3
14	25.02.2009	25.02.2009	87	90.9 g 8.2 × 6.3 × 5
15	13.03.2009	13.03.2009	Not applicable	700 g 22 × 22 × 3
16	03.04.2009	03.04.2009	43	133 g 7.0 × 8.0 × 6.0

The transportation-time was calculated out of the sample datasheet. If no time for sample extraction was marked in the sample data sheet, the earliest time for operation (07:30 am) was taken for the calculation. Therefore, the transportation-time is marked as “not applicable”.

### Tissue preparation

**Figure 4:**
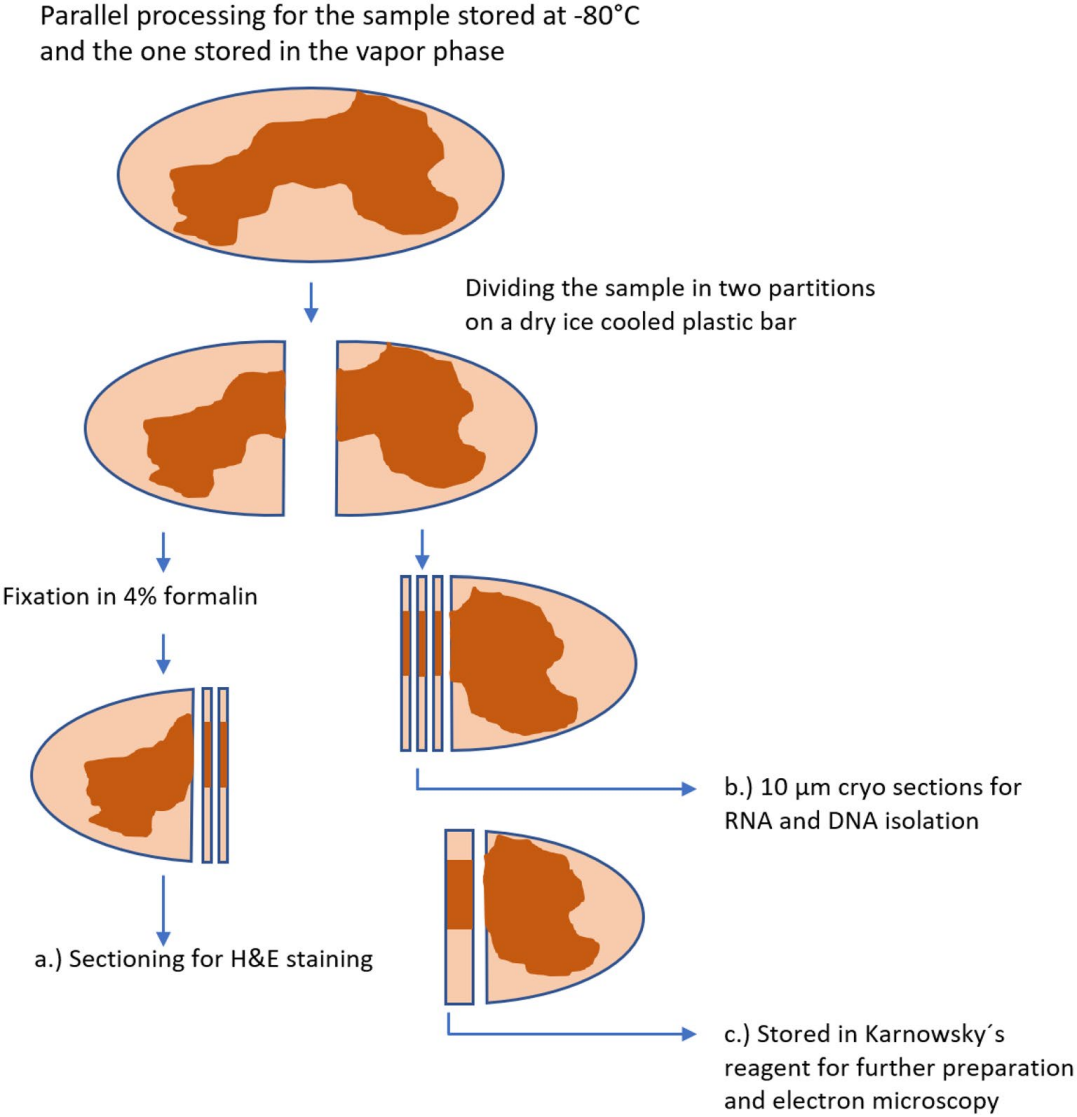
Preparation of the samples for H&E-staining, RNA- and DNA isolation and electron-microscopy. This procedure was done in parallel for each sample stored at − 80 °C as well as the sample stored in the VPLN.

First, each sample was divided into two parts on a dry ice cooled, sterile cutting board (cold work surface). One half was put into 4% formalin for 24 h fixation and subsequent H&E-staining. The other half of the sample was prepared for RNA- and DNA-extraction. For this purpose, cryo-sections of 10 µm thickness were performed with help of a cryostat at − 20 °C. To fix the sample in the cryostat, Killik-mounting medium (Bio-Optica, Milano, Italy) was used. For RNA extraction, depending on the cross-section-diameter of the sample, between 5 and 20 cryo-sections with a maximum weight of 30 mg were transferred in an 1,5 ml sample-tube and processed further as described in the next section. For DNA extraction, 5–10 cryo-sections with a maximum weight of 20 mg were transferred in an 1.5 ml sample-tube and processed further as described in the next section. After removing the sample from the cryostat, the sample was cut out of the frozen mounting medium and then transferred in Karnowsky´s reagent for electron-microscopy. Figure 4 shows a scheme of the tissue preparation.

A summary of all differences between the two samples is given in Table 4.

**Table 4:** Summary of the differences between both sample groups and the related storage protocols.

Conditions	Tumour-Bank	PATH-Biobank	Documentation
Storage	− 80 °C	VPLN (approx. − 186°)	Temperature monitoring by technical headquarter, University Hospital Regensburg
	Plastic bags	2 ml cryotubes	Storage device is documented in the storage protocol
Opening/handling	Frequent	Very few	Frequency and durations not documented
Temperature undulations	Possible More inhomogeneous temperature distribution	Less possible More homogeneous temperature distribution	No documentation of temperature fluctuations No individual sample temperature monitoring
Ice crystal formation	Higher probability Ice crystals were observed at the drawer	Lower probability No ice crystal formation could be observed	Not monitored
Sample position	Randomly located in freezer	Located in two adjacent boxes	Documented by the QM-System of the Institute of Pathology

### Analysis of the RIN^e^ and DIN-values

RNA extraction was done using the RNeasy Mini Kit (Qiagen, Hilden, Germany). After lysis of the cryosections in RLT buffer [+Dithiothreitol (DTT)] by vortexing, the kit was used according to the manual. The RIN^e^-value is based on a mathematical algorithm based on the quantitative measurement of rRNA degradation. It has a high correlation to the RIN-values that can be measured by the Agilent Bioanalyzer instead of the TapeStation^25^. RIN^e^ was measured with the Genomic RNA-ScreenTape Kit, together with the RNA ScreenTape Sample Buffer- and Ladder on the Agilent TapeStation 4200, with the corresponding Analysis-Software, Version A.02.02 (all Agilent Technologies, Santa Clara, USA).

Extraction of DNA was done out of the cryo-sections with the QIamp DNA Micro Kit (Qiagen, Hilden, Germany), as described in the kits manual. DNA was eluted with 40 µl of RNAse free water. The DIN-value was measured with the Genomic DNA ScreenTape Kit on the same Agilent TapeStation 4200 (Agilent Technologies, Santa Clara, USA).

Sectioning and extracting was done on the same day for all 32 samples. RIN^e^- and DIN-values were measured on the following day, with storing the extracted RNA and DNA at − 80 °C.

### Haematoxylin and eosin staining

For the H&E-staining, samples were fixed in buffered formalin (3.5%) for at least 24 h at room temperature followed by dehydration and paraffin embedding according to standardized and automated methods (Leica ASP 300S dehydration system, Leica Biosystems, Wetzlar, Germany; Thermo HistoStar embedding system, Thermo Scientific, Waltham, USA). After that, paraffin sections from 5 µm thickness were prepared (Microm HM 355S, Thermo Scientific, Waltham, USA) and stained with haematoxylin and eosin in a standardized and automated manner (Sakura Tissue-Tek Prisma, Sakura Finetek, Alphen aan den Rijn, The Netherlands).

### Electron-microscopy

The samples for electron-microscopical analyses were fixated in Karnowsky’s fixative for at least 72 h at room temperature. After that, 3 representative parts measuring approximately 2 × 2 × 2 mm were cut out of each sample, using a binocular microscope. The material was then embedded into Epon, using the Lynx EL Microscopy tissue processor (Electron microscopy sciences, Hatfield, USA). After embedding the samples, semi-thin-sections were generated, using the Reichert Ultracut S Microtome (Leica-Reichert, Wetzlar, Germany). Afterwards, one of the samples was chosen for further processing. Criteria for further preparation was presence of the tumour in the semi-thin-section. Ultra-thin sections (60 nm thickness) were made using the same Reichert Ultracut S Microtome. The ultra-thin-sections where transferred to ultra-sonic cleaned EM-Grids, dried and contrasted with 2%-uranyl-acetate for 10 min. After that, samples were covered with 2%-lead-citrate for 10 min. After contrasting, the samples were carefully washed using Aqua bidest. Electron-microscopy was performed using the LEO 912 AB-electron-microscope (Zeiss, Oberkochen, Germany).

### Statistical analysis

Statistical analyses were performed using R (R Core Team, open source, Version 3.5.3). The significance level was set at p < 0.05.
